# Potential IFNγ Modulation of Inflammasome Pathway in *Chlamydia trachomatis* Infected Synovial Cells

**DOI:** 10.3390/life11121359

**Published:** 2021-12-07

**Authors:** Simone Filardo, Marisa Di Pietro, Federica Frasca, Fabiana Diaco, Mirko Scordio, Guido Antonelli, Carolina Scagnolari, Rosa Sessa

**Affiliations:** 1 Section of Microbiology, Department of Public Health and Infectious Diseases, “Sapienza” University, 00185 Rome, Italy; marisa.dipietro@uniroma1.it (M.D.P.); fabiana.diaco@uniroma1.it (F.D.); rosa.sessa@uniroma1.it (R.S.); 2 Laboratory of Virology, Department of Molecular Medicine, Istituto Pasteur Italia, “Sapienza” University, 00185 Rome, Italy; federica.frasca@uniroma1.it (F.F.); mirko.scordio@uniroma1.it (M.S.); guido.antonelli@uniroma1.it (G.A.); carolina.scagnolari@uniroma1.it (C.S.)

**Keywords:** *Chlamydia trachomatis*, synovial cells, inflammasome, IFN-γ

## Abstract

Following a *Chlamydia trachomatis* infection, the host immune response is characterized by its recognition via Toll-like and Nod-like Receptors, and the subsequent activation of interferon (IFN)-γ-mediated signaling pathways. Recently, the inflammasome-mediated host cell response has emerged to play a role in the physiopathology of *C. trachomatis* infection. Here we investigated, for the first time, the interaction of IFN-γ and inflammasome in an in vitro model of *C. trachomatis*-infected primary human synovial cells. Chlamydial replication as well as the expression of caspase-1, IL-1β, as well as IL-18 and IL-6, were assayed. Our results demonstrated the inhibitory activity of IFN-γ by interfering with the inflammasome network through the downregulation of caspase-1 mRNA expression. In addition, the ability of *C. trachomatis* to hinder the inflammasome pathway favoring its intracellular survival within synovial cells, was observed. Overall, our data suggest a potential mechanism of immune evasion by *C. trachomatis* in synovial cells, that may be contested by IFN-γ.

## 1. Introduction

*Chlamydia trachomatis*, an obligate intracellular pathogen, is the leading cause of bacterial sexually transmitted diseases worldwide, with more than 130 million new cases per year [1]. *C. trachomatis* genital infection manifests as urethritis and cervicitis in women, and urethritis in men, although it is often asymptomatic, leading to chronic complications like reactive arthritis (ReA) [2,3,4].

It is well known that *C. trachomatis* infects mainly the epithelial cells of the genital tract, but it can also replicate into different cell types, including synovial cells, human placental trophoblasts, and testicular human Sertoli cells [5,6,7,8,9,10]. Following chlamydial infection, the host cell response begins with the activation of a complex network of immune receptors and their unique downstream signaling pathways, resulting in the induction of proinflammatory cytokines involved in either the elimination of *C. trachomatis* or tissue damage related to chronic inflammatory state. In particular, Toll-like receptors (TLRs), TLR2, TLR3 and TLR4, and oligomerization domain (NOD)-like receptors (NLRs), NOD1, as well as the respective downstream signaling pathways, like NFκb, IRF9 and MyD88, have been identified in the immune recognition of *C. trachomatis* in several in vivo and in vitro models [5,7,8,9,10,11].

The ability of *C. trachomatis* to infect synovial cells, as evidenced by the presence of chlamydial inclusions in human synovial fibroblasts [11], suggested its potential direct role in the development of ReA. Chlamydial inclusion is unique among intracellular pathogens and represents a chlamydial survival strategy since it provides a protected vacuole where *C. trachomatis* replicates [3,12]. Indeed, the *C. trachomatis* developmental cycle occurs within the inclusion, and it is characterized by two alternating forms, the extracellular infectious form called the elementary body (EB), and the intracellular replicative and metabolically active form called the reticulate body (RB) [12]. Under stressful conditions, *C. trachomatis* fails to complete its developmental cycle, generating persistent forms which remain inside the host cell for a long time due to their ability to evade the immune system leading to a chronic inflammatory state [13,14].

In addition to chlamydial persistent forms, the chronic inflammatory state and, hence, tissue damage might also be correlated to a dysregulated inflammasome activation, a widely studied innate immune pathway in other bacterial infections [15]. Indeed, regulated inflammasome activation usually leads to the induction of proinflammatory signaling networks with consequent bacterial infection control [16]. Inflammasomes are macromolecular protein complexes consisting mainly of an NLR family member, an adaptor apoptosis-associated speck-like protein containing a caspase recruitment domain (ASC) and caspase-1 [16]. The latter, once activated in response to infection, mediates the production of potent inflammatory cytokines like interleukin (IL)-1β and IL-18, and leads to an inflammatory form of cell death [16]. The activation of the NLRP3 inflammasome has also been involved in the pathogenesis of synovial tissue damage [17]. Specifically, the caspase-1 mediated production of IL-1β was shown to exacerbate the prodegradative mechanisms responsible for cartilage breakdown [18]. Other inflammasome related proinflammatory cytokines, like IL-18, have also been demonstrated to contribute to synovial inflammation [19].

Caspase-1 activated IL-18 is also a potent inducer of IL-1β, IL-8, and interferon (IFN)-γ [20,21], which has been demonstrated to play a key role in the clearance and protection against *C. trachomatis* by modulating a plethora of host cell signaling pathways [22,23]. Interestingly, in our recent study, the anti-chlamydial activity of IFN-γ has also been demonstrated in primary human synovial fibroblasts through the modulation of the host-cell innate immune responses, as evidenced by an increased expression of TLR2, 3 and 4, and IFN-related pathways, including GAS, STING, IRF9, ISG56, and GBP1 [11].

Given that IFN-γ and inflammasomes are two important mediators of host immune response against chlamydial infection, herein we investigated, for the first time, the interaction between IFN-y and inflammasome in an in vitro model of *C. trachomatis* infected human synovial fibroblasts. In particular, the expression of caspase-1 and the downstream inflammatory cytokines IL-1β, IL-8, IL-18 and IL-6 were determined.

## 2. Materials and Methods

### 2.1. Cell Culture and Culture Conditions

Primary human fibroblast-like synoviocytes (HFLS, 408K-o5a, Cell Applications Inc., San Diego, CA, USA) were seeded in 25 cm^2^ cell-culture flasks (angled neck, tissue-culture treated, Corning, NY, USA) and grown in Dulbecco’s Modified Eagle Medium (DMEM, with 4.5g/L glucose, L-glutamine and sodium pyruvate, Corning, NY, USA) supplemented with 15% *v*/*v* fetal bovine serum (FBS, South American Origin, Corning, NY, USA), penicillin (100 U/mL) and streptomycin (100 µg/mL) (penicillin-streptomycin Solution, 50X, Corning, NY, USA) at 37 °C in humidified atmosphere with 5% CO_2_. At 85% confluency level, cells were passaged via 2 min trypsinization (0.05% trypsin in 0.53 mM EDTA, 1X, Corning, NY, USA), and all experiments were performed using cells that were passaged for at least 4 or 5 times.

McCoy cells (ECACC, Public Health England, catalogue number 90010305, Porton Down, Salisbury, UK), were cultured in DMEM supplemented with 10% *v*/*v* FBS, at 37 °C in humidified atmosphere with 5% CO_2_.

### 2.2. Propagation and Titration of C. trachomatis

*C. trachomatis* serovar D strain UW3 (VR-885, ATCC, Manassas, VA, USA) was propagated in McCoy cells, as previously described [6]. Briefly, confluent cell monolayers, grown on 25 cm^2^ cell-culture flasks, were infected with a stock solution containing chlamydial EBs by centrifugation at 754× *g* for 30 min and then harvested by scraping after 36 to 40 h post-infection (h.p.i.). The resulting suspension was vortexed with sterile glass beads for 1 min and, after cell-debris removal by centrifugation at 250× *g* for 10 min, the supernatant, containing chlamydial EBs, was added to equal volume of 4× sucrose phosphate (4SP) buffer (0.4 M sucrose and 16 mM Na_2_HPO_4_ adjusted to pH 7.1, and filter sterilized), and stored at −80 °C.

For the titration of *C. trachomatis*, confluent cell monolayers, grown on 24-well cell-culture plates, were infected with 10-fold serial dilutions of chlamydial EB stock by centrifugation and then incubated for 48 h at 37 °C, fixed with 96% ice-cold methanol and stained with fluorescein isothiocyanate-conjugated monoclonal antibody anti-*C. trachomatis* outer membrane protein (Chlamydia Cel kit, Cellabs, Sydney, AU; catalog number KC1), as previously described [24]. The total number of *C. trachomatis* inclusion forming units (IFUs) was enumerated by counting all microscope fields using a fluorescence microscope (DM4000, Leica Microsystems GmbH, Wetzlar, Germany).

### 2.3. Infection of Human Synovial Cells with C. trachomatis and Pretreatment with IFN-γ

Human synovial cells were grown on 24-well cell-culture plates or on 25 cm^2^ cell-culture flasks. Upon confluence, cell monolayers were either pre-incubated with IFN-γ at a concentration of 10^3^ U/mL in DMEM with 15% FBS, or pre-incubated in DMEM with 15% FBS alone, at 37 °C in humidified atmosphere with 5% CO_2_, as previously described [11]. After 24 h, cell monolayers were washed with PBS, infected with *C. trachomatis* at a multiplicity of infection (MOI) of 1.0 as above described, and then incubated at 37 °C for 6, 18, 24 and 36 h.p.i. At each time-point, the cell monolayers on 24-well cell-culture plates were either fixed with ice-cold methanol and stained as above described, for the determination of *C. trachomatis* infection efficiency via fluorescence microscopy or harvested and stored at −80 °C for the infectivity assay. Infected cell monolayers on 25 cm^2^ cell-culture flasks were harvested by scraping, pelleted by centrifugation at 10,000× *g* for 10 min, and stored at −80 °C for mRNA extraction and real-time RT-PCR assay. Cell supernatants were also aliquoted and stored at −80 °C for the determination of protein levels via ELISA.

### 2.4. Efficiency of C. trachomatis Infection in Human Synovial and McCoy Cells

The efficiency of *C. trachomatis* infection in human synovial and McCoy cells was investigated by infecting confluent cell monolayers grown on coverslips in 24-well cell-culture plates with *C. trachomatis* at a MOI of 1.0 as above described. After 36 h incubation, the number of chlamydial IFUs was enumerated by counting all microscope fields via a fluorescence microscope.

### 2.5. Growth Curve of C. trachomatis in Human Synovial Cells

The growth curve of *C. trachomatis* in human synovial cells was performed infecting confluent cell monolayers, grown on 24-well cell-culture plates, with *C. trachomatis* at a MOI of 1.0. At 6, 18, 24 and 36 h.p.i., infected cells were harvested and stored at −80 °C. To assess the infectivity at each time-point, *C. trachomatis* EBs suspensions were titrated as above described.

### 2.6. TaqMan-Based Real-Time RT-PCR Assay

Quantitative real-time PCR for caspase-1, IL-1β, IL-8, IL-18 and IL-6 was carried out with the LightCycler 480 instrument (Roche, Basel, Switzerland). Briefly, at 6, 18 and 24 h.p.i., total RNA was extracted using the RNeasy Plus Universal Tissue Mini Kit (Invitrogen, Carlsbad, CA, USA). RNA extracts were reverse transcribed using the High-Capacity cDNA Reverse Transcription Kit (Applied Biosystems, Woburn, MA, USA), according to the manufacturer’s instruction. Primers and probes for each gene were added to the Probes Master Mix (Roche, Basel, Switzerland) at 500 and 250 nM, respectively, in a final volume of 20 μL. The housekeeping gene β-glucuronidase (GUS) was used as an internal control. GUS was selected as a good candidate for the housekeeping gene in our experimental setting, because it was constantly expressed in synovial cells after *C. trachomatis* or IFN stimulation. Gene expression values were calculated by the comparative 2−ΔCt methods. The primers and probe were assayed on demand and were purchased from Integrated DNA Technologies (IDT), Clear Creek, IA, USA. The list of primers and probes is as follows: IFNAR1: Hs.PT.58.20048943; IFNAR2: Hs.PT.58.1621113; IFNGR1: Hs.PT.58.20918191; IFNGR2: Hs.PT.58.38961914; IL-1β: Hs.PT.58.1518186; caspasi1: Hs.PT.59a.22997425.g; IL-6: Hs.PT.58.40226675; IL-18: Hs.PT.58.25675872; IL-8: Hs.PT.58.39926886.g.

### 2.7. ELISA Assay

At 6, 18 and 24 h.p.i., the supernatants collected from infected human synovial cells were analyzed for IL-1β, IL-8, IL-18 and IL-6 using the specific FineTest ELISA kits (Wuhan Fine Biotech Co., Ltd., Wuhan, China; catalog numbers EH0185, EH0205, EH0011 and EH0201, respectively), according to the manufacturer’s instructions. The detection limit for each cytokine is as follows: Il-1β, 2.344 pg/mL; Il-8, 18.75 pg/mL; Il-18, 9.375 pg/mL and IL-6, 2.813 pg/mL.

### 2.8. Caspase Inhibition in Human Synovial Cells

Confluent synovial monolayers, grown on coverslips in 24-well cell-culture plates, were either pre-incubated with IFN-γ at a concentration of 10^3^ U/mL, or in DMEM with 15% FBS alone, at 37 °C in humidified atmosphere with 5% CO_2_. After 24 h incubation, cell monolayers were infected with *C. trachomatis* at a MOI of 1.0 as above described. At 6 h.p.i., the pan-caspase inhibitor Z-VAD-FMK (Enzo Life Sciences, Inc., Farmingdale, NY, USA) was added to the infected cell monolayers at a concentration of 20 µM. At 36 h.p.i., cells were fixed with ice-cold methanol and stained as above described, and the number of chlamydial IFUs was enumerated by counting all microscope fields via a fluorescence microscope. At the same time, the cytotoxic effect of the pan-caspase inhibitor on the human synovial cell line at the concentration of 20 µM was tested via MTT assay and viable cell count, as previously described [25].

### 2.9. Statistical Analysis

All values were expressed as mean ± standard deviation (SD) of three replicates from three independent experiments. After assessing data normality via Shapiro-Wilk test, the comparison of means was either performed by using a two-tailed student *t*-test for independent samples or a Mann–Whitney U test for non-parametric data distributions. Two-way ANOVA was performed for the analysis of variance. All statistical calculations were performed in Microsoft Excel software (version 2110), by using the Real Statistics Resource Pack (release 7.9.1, https://www.real-statistics.com, accessed on 1 July 2021). A value of *p* < 0.05 was considered statistically significant.

## 3. Results

### 3.1. C. trachomatis Productively Infects Primary Human Synovial Cells

**Figure 1:**
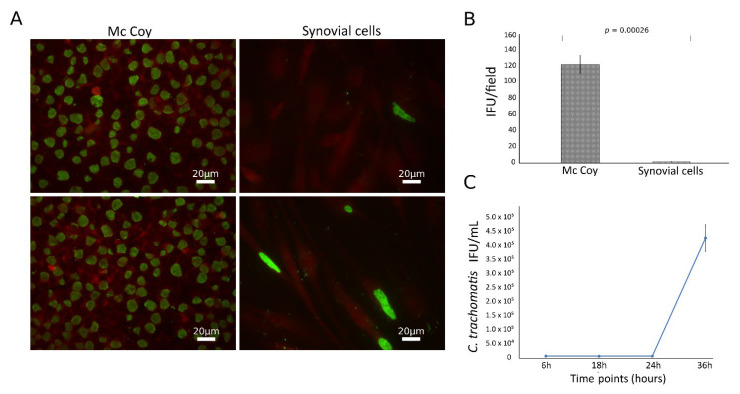
Growth characteristics of *C. trachomatis* in synovial cells. Synovial and McCoy cells were infected with *C. trachomatis* (MOI 1.0), and, at 36 h.p.i, chlamydial inclusions were counted by immunofluorescence staining; (**A**) representative micrographs of *C. trachomatis* inclusions in synovial and McCoy cells; (**B**) *C. trachomatis* infection efficiency in human synovial and McCoy cells. (**C**) Kinetics of replication of *C.* *trachomatis* in synovial cells. Infected synovial cells (MOI 1.0) were harvested at 6, 18, 24 and 36 h.p.i. and inoculated onto McCoy cells. After 36 h.p.i., the recoverable viable *C. trachomatis* bacteria were quantitated and expressed as IFU/mL.

*C. trachomatis* showed the ability to productively infect the primary human synovial cells, as shown by the presence of the typical green-fluorescent inclusions after staining with FITC-conjugated mouse monoclonal antibody anti-chlamydial MOMP (Figure 1A). However, the infection rate was significantly lower than that in McCoy cells, the gold standard for *Chlamydia* laboratory culture, with a synovial to McCoy cells ratio of 0.013 (*p* = 0.00026, Figure 1B). Chlamydial inclusion morphology was also different in synovial as compared to McCoy cells, where inclusions had similar size and shapes; by contrast, in synovial cells, inclusions presented a heterogeneous morphology, as shown in Figure S1. The presence of *C. trachomatis* inclusions in synovial cells was further confirmed via staining with anti-*C. trachomatis* LPS monoclonal antibody (Figure S2).

To characterize the growth phenotype of *C. trachomatis* in primary human synovial cells, we sampled multiple time points across the duration of the chlamydial developmental cycle for determining chlamydial EB infectivity. The infectivity growth curve showed a relatively long eclipse period for *C. trachomatis* in human synovial cells, with infectious EBs appearing at 36 h postinfection (Figure 1C). No persistent forms have been identified in the experimental conditions studied, as evidenced by no reduction of inclusion via infectivity assay (Figure 1C).

### 3.2. C. trachomatis Infection of Human Synovial Cells Upregulates the Expression of IL-1β and Caspase-1 Genes Involved in the Host-Cell Inflammasome Pathway

**Figure 2:**
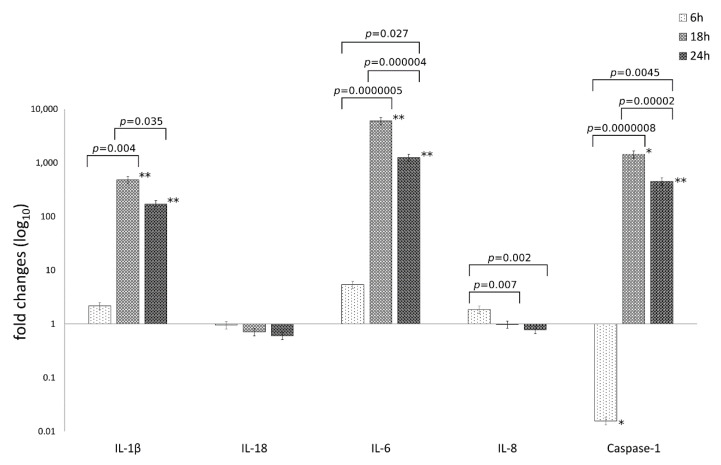
Relative mRNA expression of IL-1β, IL-18, IL-6, IL-8 and caspase-1 following *C. trachomatis* infection of synovial cells. Results are expressed as fold changes, as compared to uninfected cells, of mRNA expression levels at 6, 18 and 24 h.p.i., via reverse transcription real-time PCR. *, *p* < 0.05; **, *p* < 0.01.

The mRNA expression levels of caspase-1 and the inflammatory mediators IL-1β, IL-6, IL-18, IL-8 are reported in Figure 2.

The analysis of variance of inflammatory mediator levels in human synovial cells following *C. trachomatis* infection as compared to uninfected cells, showed statistically significant differences related to the type of inflammatory mediator (IL-1β, IL-6, IL-18, IL-8 and caspase-1) or the time postinfection (6, 18 and 24 h.p.i.) as well as the interaction between these two factors (*p* = 2.69 × 10^−21^, *p* = 1.73 × 10^−14^ and *p* = 8.70 × 10^−17^, respectively).

Particularly, *C. trachomatis* infection of human synovial cells led to a more than 100-times increase in the mRNA expression levels of IL-1β, IL-6 and caspase-1 genes at 18 and 24 h.p.i. (*p* < 0.05), while no increase was observed for the inflammatory mediators IL-8 and IL-18 (Figure 2).

Interestingly, IL-1β, IL-6 and caspase-1 mRNA levels increased after 6 h.p.i. with a peak expression at 18 h.p.i. (*p* < 0.00001) and consistently high levels at 24 h.p.i.; IL-6 showed the highest levels as compared to the other inflammatory mediators (*p* < 0.01, Figure 2).

### 3.3. IFNγ Pretreatment of Human Synovial Cells Modulates the Host Cell Response against C. trachomatis Infection

**Figure 3:**
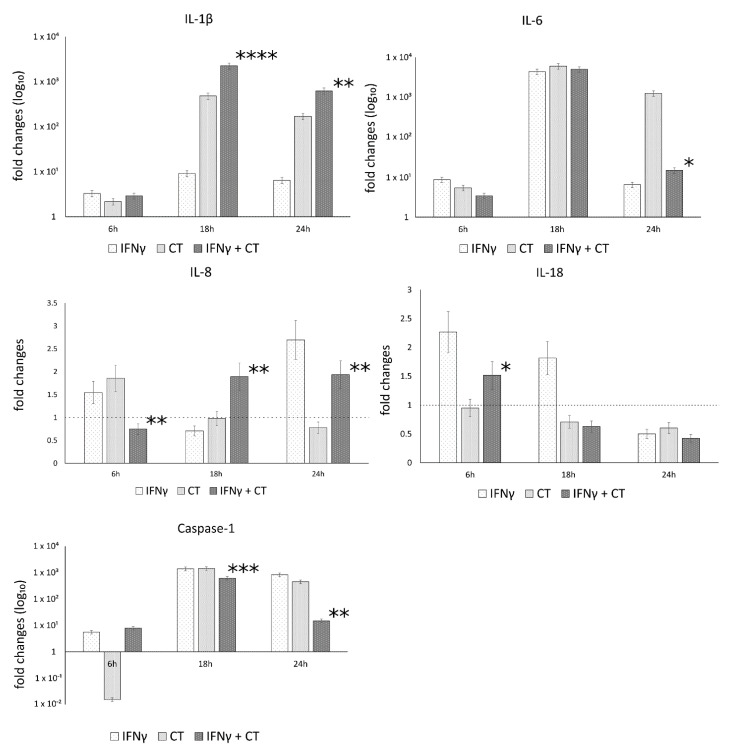
IFN-γ effects on the relative mRNA expression of IL-1β, IL-18, IL-6, IL-8 and caspase-1. *C. trachomatis* infected synovial cells pretreated with IFN-γ as well as untreated or uninfected cells were assayed via reverse transcription real-time PCR. * *p* < 0.05, ** *p* < 0.01, *** *p* < 0.0001 and **** *p* < 0.00001 vs. *C. trachomatis* infected cells.

The mRNA expression levels of caspase-1 and the inflammatory mediators IL-1β, IL-18, IL-6, IL-8, in the presence or absence of IFN-γ pretreatment of human synovial cells, are reported in Figure 3.

The analysis of variance between IL-1β, IL-18, IL-6, IL-8 and caspase-1 levels, showed statistically significant differences related to the infection conditions or the time after infection (6, 18 and 24 h.p.i.) as well as the interaction between these two factors (Table S1).

In *C. trachomatis*-infected synovial cells, the pretreatment with IFN-γ significantly decreased the mRNA expression levels of IL-6 and Caspase-1 genes at later time points during the chlamydial developmental cycle (18 and 24 h.p.i. for caspase-1 and 24 h.p.i. for IL-6, *p* < 0.000001 and *p* < 0.05, respectively), as evidenced by Figure 3. Conversely, the pretreatment with IFN-γ significantly increased the expression of IL-1β and IL-8 genes at both 18 and 24 h.p.i. (*p* < 0.01, Figure 3). IL-18 showed only a modest increase at 6 h.p.i. (*p* < 0.05, Figure 3).

### 3.4. IFN-γ Pretreatment in C. trachomatis Infected Human Synovial Cells Modulates IFN Receptor Gene Expression

Following the observation that IFN-γ pretreatment of chlamydia-infected human synovial cells modulated the gene expression of IL-1β and caspase-1, we explored whether IFNγ may also regulate the expression of IFN-I and IFN-II receptor genes, namely the receptor chains IFNαR1 and IFNαR2, as well as IFNγR1 and IFNγR2, potentially exerting positive feedback on its own signaling pathways.

**Figure 4:**
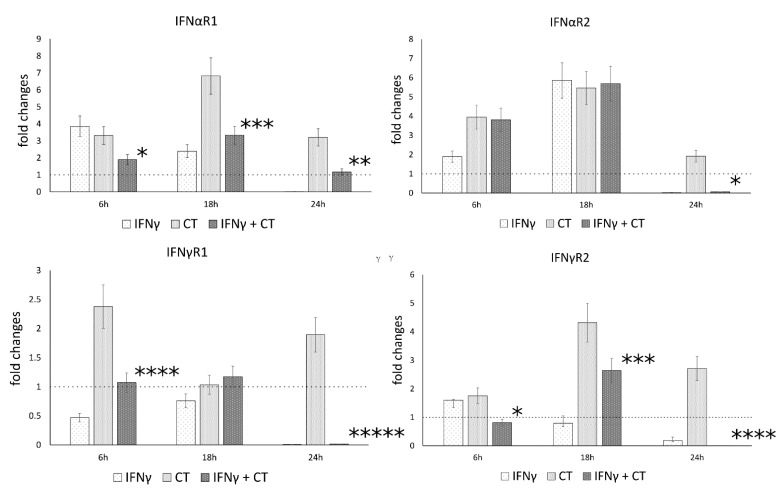
IFN-γ effects on the relative mRNA expression of IFNαR1, IFNαR2, IFNγR1 and IFNγR2 receptor chains. *C. trachomatis* infected synovial cells pretreated with IFNγ as well as untreated or uninfected cells were assayed via reverse transcription rt-PCR. * *p* < 0.05, ** *p* < 0.01, *** *p* < 0.001, **** *p* < 0.0001 and ***** *p* < 0.00001 vs. *C. trachomatis* infected cells.

Our results showed that *C. trachomatis* infection induced an overall increase in the mRNA levels of type-I IFN receptor at all time points (6, 18 and 24 h.p.i.), with the highest mRNA expression at 18 h.p.i. for the IFNαR1 and IFNαR2 (*p* < 0.001) (Figure 4). Conversely, the mRNA expression levels of type-I and type-II IFN receptor genes significantly decreased after IFNγ pretreatment of chlamydia-infected human synovial cells at either 18 h.p.i. (IFNαR1 and IFNγR2, *p* < 0.001) or 24 h.p.i. (IFNαR1 and IFNαR2, as well as IFNγR1 and IFNγR2, *p* < 0.01, *p* < 0.05, *p* < 0.000001 and *p* < 0.000001, respectively, Figure 4).

### 3.5. IFN-γ Pretreatment in C. trachomatis-Infected Human Synovial Cells Increases Inflammatory Cytokine Production

Since an increase in caspase-1, IL-1β and IL-6 gene expression levels was observed following *C. trachomatis* infection of human synovial cells, we, then, proceeded to analyze their related protein levels.

**Figure 5:**
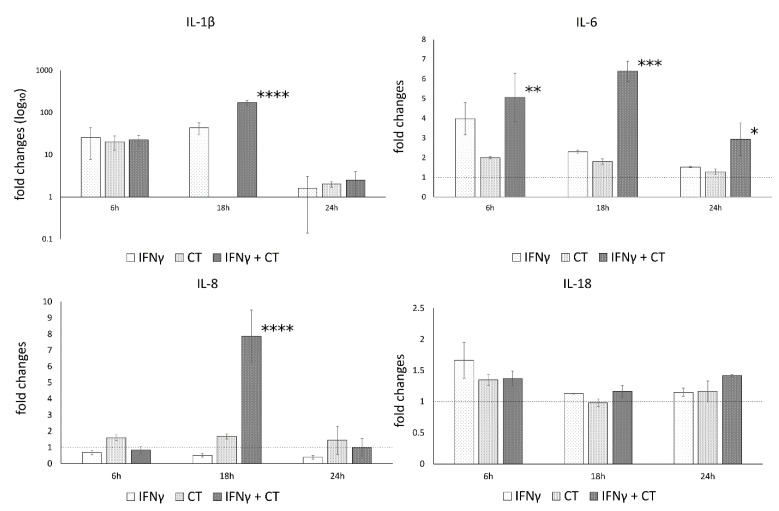
IFN-γ effects on the protein levels of IL-1β, IL-6, IL-8 and IL-18. *C. trachomatis*-infected synovial cells pretreated with IFN-γ as well as untreated or uninfected cells were assayed via ELISA. * *p* < 0.05, ** *p* < 0.001, *** *p* < 0.0001 and **** *p* < 0.000001 vs. *C. trachomatis* infected cells.

In contrast to the mRNA expression data, no statistically significant increase in the related protein levels of the cytokines IL-1β, IL-6, IL-18 and IL-8 was observed following *C. trachomatis* infection of human synovial cells, as evidenced in Figure 5.

On a different note, IFN-γ pretreatment led to a statistically significant increase in the protein levels of IL-1β, IL-6 and IL-8 at 6 or 18 h.p.i., as shown in Figure 5, mirroring the mRNA expression data, while no increase was observed for IL-18.

### 3.6. The Anti-Chlamydial Activity of IFN-γ Is Correlated to the Inhibition of Caspases

To investigate whether inflammasome related caspases played a role in *C. trachomatis* replication and was involved in the anti-chlamydial activity of IFN-γ, chlamydia infected human synovial cells were exposed to the pan-caspase inhibitor.

**Figure 6:**
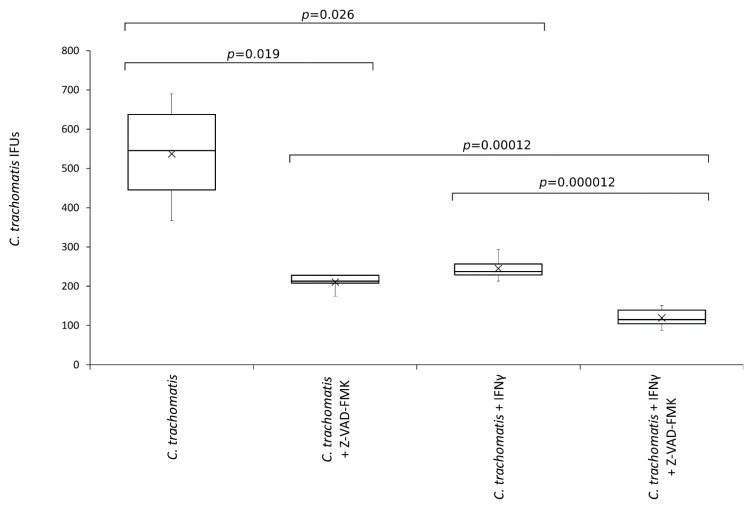
Effects of pan-caspase inhibitor on *C. trachomatis* IFUs. IFN-γ pretreated and untreated synovial cell monolayers were infected with *C. trachomatis* at a MOI of 1.0 and exposed to the pan-caspase inhibitor Z-VAD-FMK (20 µM) at 6 h.p.i. The number of chlamydial IFUs was counted by fluorescence microscopy.

As shown in Figure 6, the number of *C. trachomatis* IFUs significantly decreased after the treatment with the pan-caspase inhibitor (*p* < 0.05). Interestingly, the reduction of chlamydia replication following the inhibition of caspases was comparable to that caused by IFN-γ pretreatment of synovial cells (−60.8% vs. −54.4%, respectively, *p* = NS). Lastly, the anti-chlamydial activity of IFN-γ was significantly potentiated by the inhibition of caspases (*p* < 0.0001).

Of note, the treatment with pan-caspase inhibitor had no cytotoxic effect on the synovial cells, as evidenced by MTT assay and viable cell count (Figure S3), showing, hence, a specific activity toward *C. trachomatis*.

## 4. Discussion

The main results of our study show that: (i) *C. trachomatis* may affect the canonical inflammasome pathway, productively infecting primary human synovial cells; (ii) IFN-γ inhibits *C. trachomatis* replication by partly acting on the host-cell immune response, most likely via the regulation of the inflammasome network.

In our chlamydial infection model based on primary human synovial cells, we demonstrated that *C. trachomatis* infection might induce the canonical inflammasome pathway promoting caspase-1 gene expression.

The activation of the inflammasome pathway is key to limiting infection by several invading bacterial pathogens, but it requires tight regulation in order to prevent inflammation and immunopathology, since it typically leads to the production of the most potent proinflammatory cytokines (IL-1β and IL-18) and the subsequent induction of inflammatory cell death [16].

In our study, unconventionally, inflammasome-mediated caspase-1 gene expression seemed to play an important role in intracellular growth of *C. trachomatis* in synovial cells, as suggested by caspase inhibition. This leads to the hypothesis that *Chlamydia* employed an evasion strategy against the host cell response, as also hinted at by the absent production of IL-1β and IL-18, observed in our study. In support of the involvement of caspase-1 in the replication phase of *C. trachomatis*, a significant reduction in the chlamydial inclusion number was observed after the treatment with a pan-caspase inhibitor. Consistent with this observation, blocking caspase-1 with an inhibitor in fibroblasts makes them less susceptible to *C. trachomatis* infection [26]. Similarly, in cervical epithelial cells, inflammasome-dependent caspase-1 activation was demonstrated to contribute to chlamydial growth and did not lead to IL-1β production [27].

By contrast, in other cell types, such as monocyte-macrophages and trophoblasts, the activation of inflammasome after *C. trachomatis* infection was demonstrated to mediate IL-1β production in a process requiring caspase-1 activation [9,28,29,30,31].

Although the basis for caspase-1 stimulation of chlamydial infection is not well defined [32] and remains poorly characterized in our human synovial cell model, we found that exposure of *C. trachomatis*-infected cells to IFN-γ may modulate the canonical inflammasome pathway by reducing the gene expression of caspase-1. These findings support our previous work on anti *C. trachomatis* activity of IFN-γ and yield novel insights into mechanisms by which this cytokine could interfere with its replication [11]. Previous studies have shown that caspase-1 can be upregulated by IFN-γ in distinct cell lines [33,34,35]. However, it has been also proposed that IFN-inducible proteins could act also as negative regulators for inflammasome activation [36], underlining the increasing complexity of this regulatory network. In this regard, IFN-γ not only inhibits *C. trachomatis* metabolic growth favoring the depletion of tryptophan [37], but also interferes with several host immune pathways [38], like caspase-1 downregulation, as evidenced by our findings. Indeed, IFN-γ knockout mice exhibited Th2-associated delayed-type hypersensitivity and the inflammatory cells failed to localize and control the chlamydial infection, indicating that IFN-γ exerts beneficial effects for the host’s defense against *Chlamydia* infection [39]. In this study, we further demonstrated that IFN-γ led to increased inflammation, as compared to *C. trachomatis* infection alone, characterized by IL-1β, IL-8 and IL-6. These proinflammatory cytokines are typically associated to cell damage [40], although it remains unknown whether this IFN-γ-mediated immunological effect on human synovial cells is helpful or harmful during *C. trachomatis* infection. However, both IL-1β and IL-8 have been shown to actively participate with other cytokines in the systemic inflammatory response during *C. trachomatis* infection [41,42]. Moreover, the expression of these cytokines can be tightly regulated by IFN-γ through several positive and negative feedback loops [43,44], suggesting that this cytokine drives other inflammatory pathways, partly contrasting the potential evasion of the host-cell immune response following *Chlamydia* infection. We also found a large discrepancy between mRNA transcript levels (Figure 2) and protein expression levels (Figure 5), especially IL-1β and IL-6. The lack of correlation of the cytokine mRNA to protein levels of IL-1b and IL-6 was not unexpected as regulation of the actual protein level is likely more complex than a direct relationship to the amount of mRNA and the rate of translation and thus the amount of mRNA does not necessarily directly correlate with the level of protein expressed. Indeed, evidence in the literature indicates that multiple post-transcriptional or translational mechanisms are involved in regulating the synthesis of these two cytokines [45,46,47,48,49]. In this regard, it is also known that cytokine mRNA decay is tightly regulated at the post-transcriptional level through cis- or trans-acting elements [50].

A limitation of our study is that transcript levels are not sufficient to predict caspa-se-1 activation levels. Caspase-1 activation occurs via proteolytic cleavage of pro-caspase molecules, leading to relatively short-lived protein products that can be detected in model systems by Western blot analysis. However, the data generated by this in vitro analysis may not reflect the actual in vivo effects caused by *C. trachomatis* infection and/or IFN-γ response on caspase-1 activation. Alternatively, detection of the correct fragments of unseparated and cleaved IL-1b and/or IL-18 might show caspase-1 activity. We addressed the question of caspase-1 involvement in the synovial cell model of *C. trachomatis* infection and in vitro regulation by IFN-γ through testing a potentially longer inflammation-related signature, the transcriptional regulation of caspase-1 mRNA. However, these results advise caution in overinterpreting the significance of the changes found in our in vitro model of *C. trachomatis*-infected primary human synovial cells.

Of note, we found that IFN-γ decreased the expression of the type-II IFN receptors, whereas *C. trachomatis* infection induced an overall increase in the expression of all the IFN receptor chains. The latter might be used to render these cells more resistant to the antiproliferative activity of IFN-γ; in this context, it has been shown that the reduced levels of the IFNγ-R2 subunit can allow Th1 cells to proliferate during IFN-γ signaling [51]. On the other hand, Th2 cells, that do not synthetize IFN-γ, produce increased levels of the IFNγ-R2 chain, rendering them particularly responsive to the antiproliferative activity of IFN-γ [51]. Thus, the decrease in the expression of IFNγ-R1 and R2 chains on the synovial cells observed after IFN-γ pretreatment could be a plausible regulatory mechanism by which these cells are desensitized in response to this proinflammatory cytokine, potentially reducing long-term tissue damage.

## 5. Conclusions

In conclusion, our data highlight the ability of IFN-γ to interfere with the inflammasome network for *C. trachomatis* infection control in synovial cells. In fact, in the absence of IFNγ, *C. trachomatis* might evade the host cell immune response affecting the inflammasome pathway. On the downside, the presence of IFN-γ might contribute to chronic inflammation by increasing proinflammatory cytokine production, potentially harmful for host cells. However, both the mechanism and functional relevance of IFN-γ and the inflammasome network in *C. trachomatis* deserve more attention, and further studies are needed to better address the effects of *C. trachomatis* and IFN-γ response on caspase-1 activation in primary human synovial cells.

## Data Availability

Data is contained within the article or supplementary material.

## Supplementary Materials

The following are available online at https://www.mdpi.com/article/10.3390/life11121359/s1, Table S1: Analysis of variance (two-factor ANOVA) for the mRNA expression levels of selected inflammatory genes in different times after infection as well as infection conditions, Table S2: Analysis of variance (two-factor ANOVA) for the protein levels, via ELISA, of selected cytokines in different times after infection and infection conditions. Figure S1: Fluorescence microscopy pictures of *C. trachomatis* inclusion morphology in primary human synovial cells. Cells were infected with *C. trachomatis* at a MOI of 1.0 and stained with a FITC-conjugated anti-*C. trachomatis* MOMP monoclonal antibody (Chlamydia Cell kit, Cellabs, Sydney, AU), as described in Materials and Methods. All pictures were taken at 36 h.p.i. Figure S2: Fluorescence microscopy pictures of *C. trachomatis* inclusions in primary human synovial cells. Cells were infected with *C. trachomatis* at a MOI of 1.0 and stained with the primary antibody anti-*C. trachomatis* LPS monoclonal antibody (Mab29, CT601, Chlamydia Biobank, UK) combined with a goat anti-Mouse IgG antibody conjugated with Alexa Fluor Plus-488 (Invitrogen, Thermo Fisher Scientific, USA). Wheat Germ Agglutinin conjugated with Alexa Fluor-594 (Invitrogen, Thermo Fisher Scientific, USA) was used for counterstaining cell monolayer. All pictures were taken at 36 h.p.i. Figure S3: Effect of pan-caspase inhibitor Z-VAD-FMK on primary human synovial cell viability. (a) MTT assay; (b) Viable cell count assay.
