# Supplementary material for: Potential IFNγ Modulation of Inflammasome Pathway in Chlamydia trachomatis Infected Synovial Cells
Source: Life (Basel). 2021 Dec 7;11(12):1359. doi: 10.3390/life11121359 (PMC8707573; doi:10.3390/life11121359)

Table S1. Analysis of variance (two-factor ANOVA) for the mRNA expression levels of selected inflammatory genes in different times after infection as well as infection conditions.

		Sum of Squares	***Mean squares***	***F-ratio***	***p-value***
**IL-1β**	Variable A	3022764.84	1511382.42	95.90	*0.000001*
	Variable B	2646493.40	1323246.70	83.96	*0.000002*
	A x B Interaction	3007107.31	751776.83	47.70	*0.000005*
**IL-18**	Variable A	2.11	1.06	28.90	*0.0001*
	Variable B	3.42	1.71	46.70	*0.00002*
	A x B Interaction	1.41	0.35	9.62	*0.003*
**IL-6**	Variable A	3111663.72	1555831.86	7.00	*0.01*
	Variable B	95960684.61	47980342.30	215.91	*0.00000002*
	A x B Interaction	1770463.49	442615.87	1.99	**N.S.**
**IL-8**	Variable A	0.63	0.32	5.02	*0.03*
	Variable B	1.16	0.58	9.22	*0.01*
	A x B Interaction	5.93	1.48	23.47	*0.0001*
**Caspase-1**	Variable A	930357.71	465178.86	32.12	*0.0001*
	Variable B	4016302.96	2008151.48	138.66	*0.0000002*
	A x B Interaction	574740.66	143685.17	9.92	*0.002*

Variable A, hours post infection (6, 18 and 24 h.p.i.); Variable B, infection conditions (Synovial cells infected with *C. trachomatis*, *Chlamydia* infected cells pre-treated with IFNγ and uninfected cells pre-treated with IFNγ); N.S., Not Significant.

Table S2. Analysis of variance (two-factor ANOVA) for the protein levels, via ELISA, of selected cytokines in different times after infection and infection conditions.

		Sum of Squares	***Mean squares***	***F-ratio***	***p-value***
**IL-1β**	Variable A	10678.46	5339.23	44.00	*0.00002*
	Variable B	15530.99	7765.49	63.99	*0.000005*
	A x B Interaction	20855.96	5213.99	42.97	*0.00001*
**IL-18**	Variable A	0.09	0.047	2.77	**N.S.**
	Variable B	0.42	0.21	12.43	*0.003*
	A x B Interaction	0.16	0.04	2.39	**N.S.**
**IL-6**	Variable A	30.51	15.25	43.15	*0.00002*
	Variable B	11.28	5.64	15.94	*0.001*
	A x B Interaction	7.73	1.93	5.46	*0.02*
**IL-8**	Variable A	22.11	11.05	25.64	*0.0002*
	Variable B	22.35	11.17	25.91	*0.0002*
	A x B Interaction	42.28	10.57	24.51	*0.0001*

Variable A, hours post infection (6, 18 and 24 h.p.i.); Variable B, infection conditions (Synovial cells infected with C. trachomatis, Chlamydia infected cells treated with IFNγ and uninfected cells treated with IFNγ); N.S., Not Significant.

Figure S1. Fluorescence microscopy pictures of *C. trachomatis* inclusion morphology in primary human synovial cells. Cells were infected with *C. trachomatis* at a MOI of 1.0 and stained with a FITC-conjugated anti-*C. trachomatis* MOMP monoclonal antibody (Chlamydia Cell kit, Cellabs, Sydney, AU), as described in Materials and Methods. All pictures were taken at 36 h.p.i.

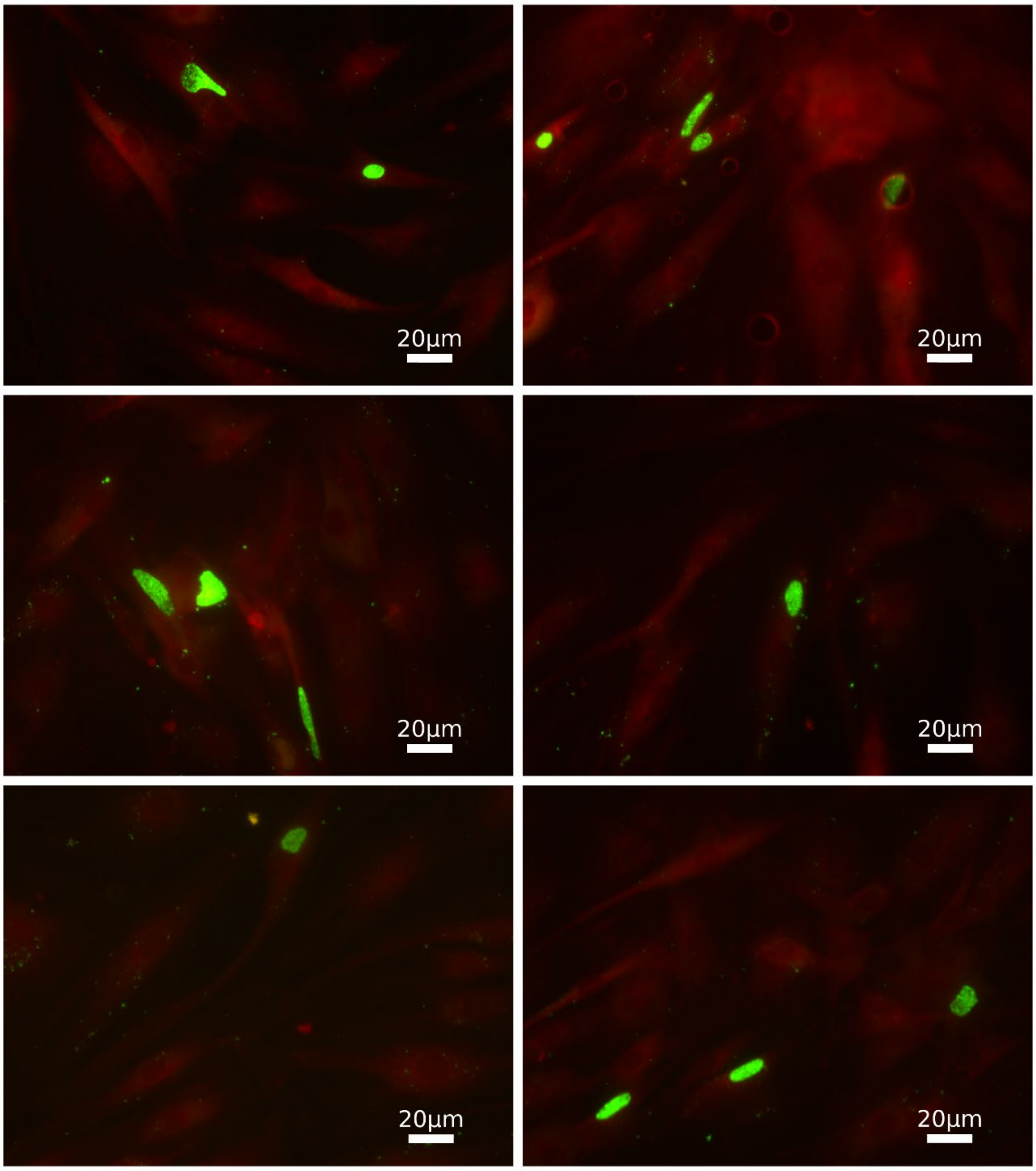

Figure S2. Fluorescence microscopy pictures of *C. trachomatis* inclusions in primary human synovial cells. Cells were infected with *C. trachomatis* at a MOI of 1.0 and stained with the primary antibody anti-*C. trachomatis* LPS monoclonal antibody (Mab29, CT601, Chlamydia Biobank, UK) combined with a goat anti-Mouse IgG antibody conjugated with Alexa Fluor Plus-488 (Invitrogen, Thermo Fisher Scientific, USA). Wheat Germ Agglutinin conjugated with Alexa Fluor-594 (Invitrogen, Thermo Fisher Scientific, USA) was used for counterstaining cell monolayer. All pictures were taken at 36 h.p.i.

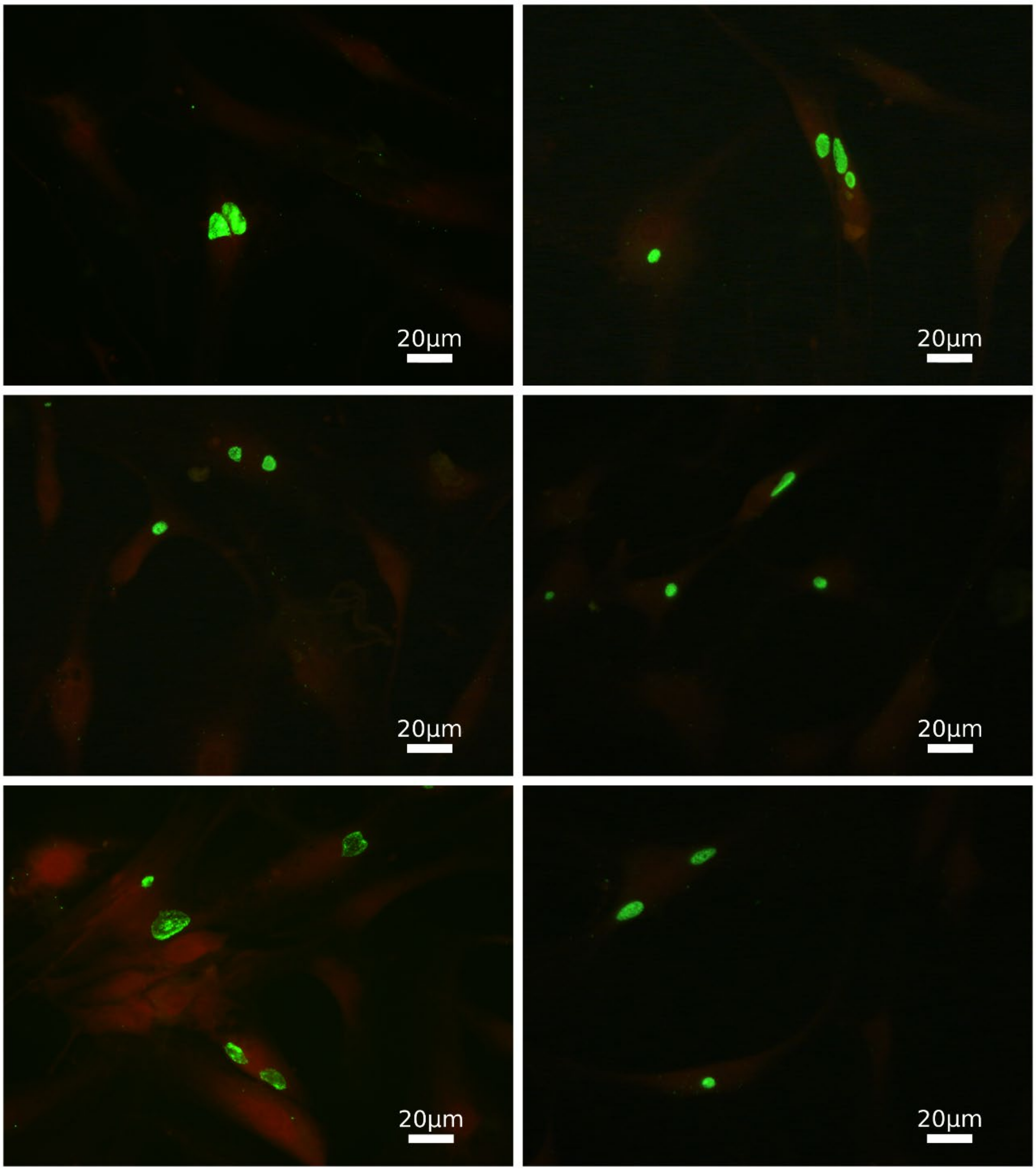

Figure S3. Effect of pan-caspase inhibitor Z-VAD-FMK on primary human synovial cell viability. (**a**) MTT assay; (**b**) Viable cell count assay.

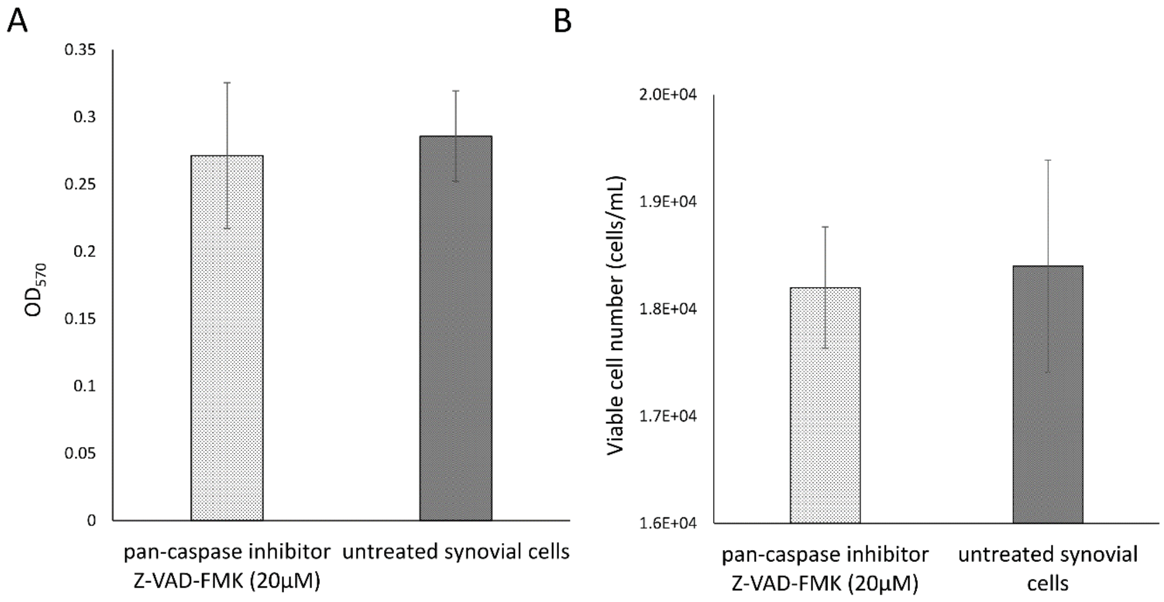

Supplement: Supplementary file 1 [file life-11-01359-s001.zip › Supplementary_materials.pdf]
