# Supplementary figures and images for: Potential IFNγ Modulation of Inflammasome Pathway in Chlamydia trachomatis Infected Synovial Cells
Source: Life (Basel). 2021 Dec 7;11(12):1359. doi: 10.3390/life11121359 (PMC8707573; doi:10.3390/life11121359)

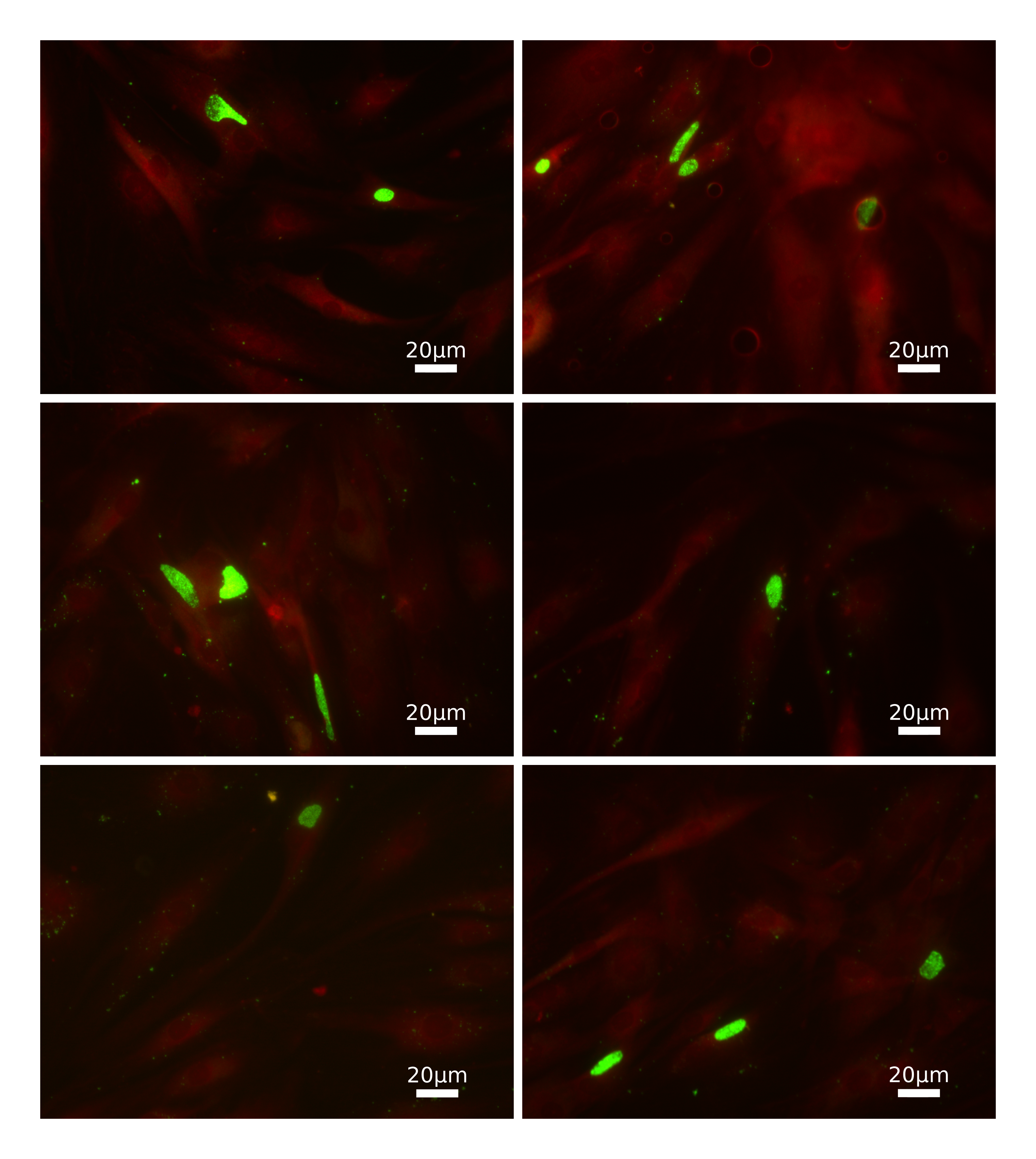

Supplement: Supplementary file 1 [file life-11-01359-s001.zip › Figure S1.tif]

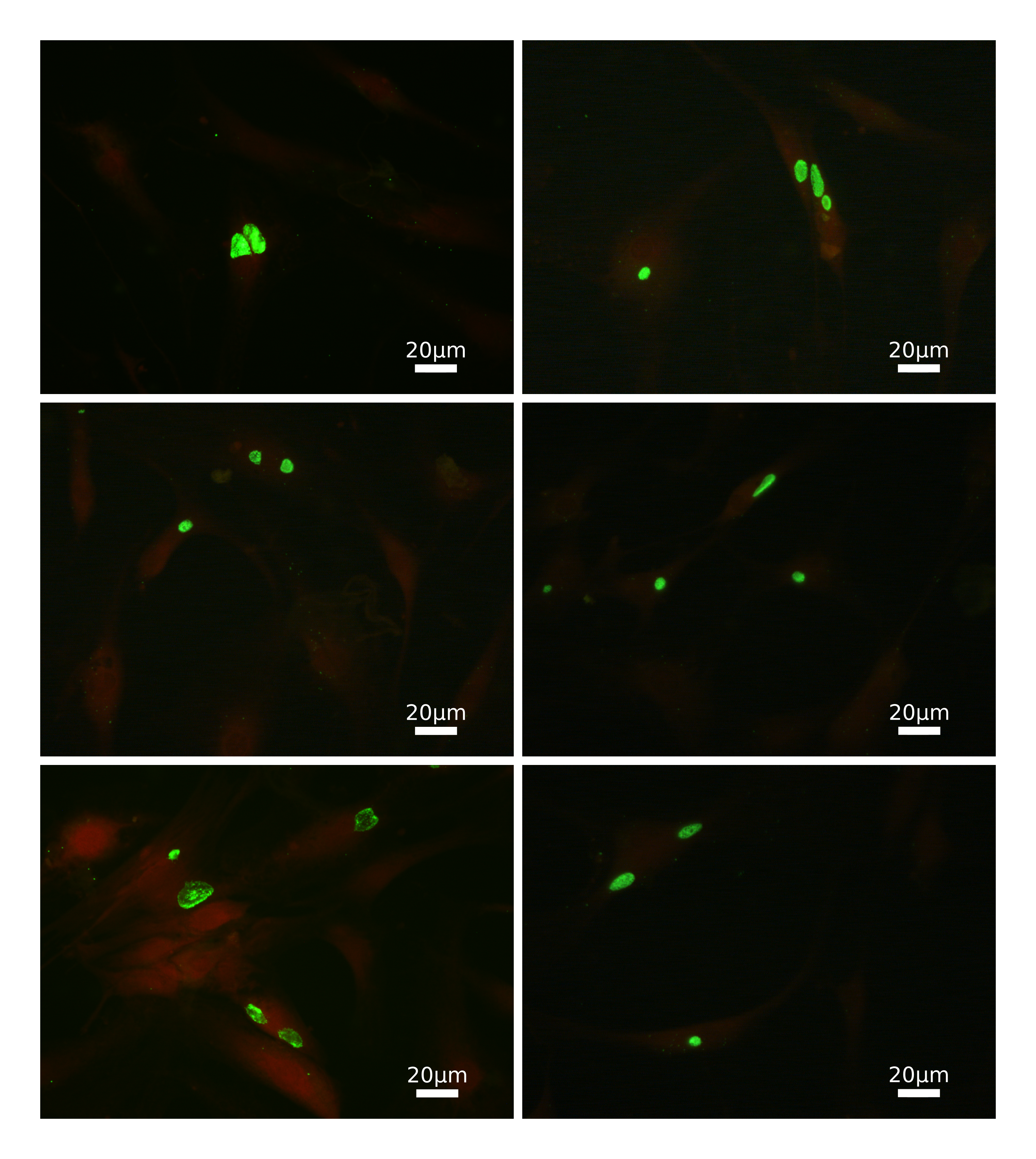

Supplement: Supplementary file 1 [file life-11-01359-s001.zip › Figure S2.tif]

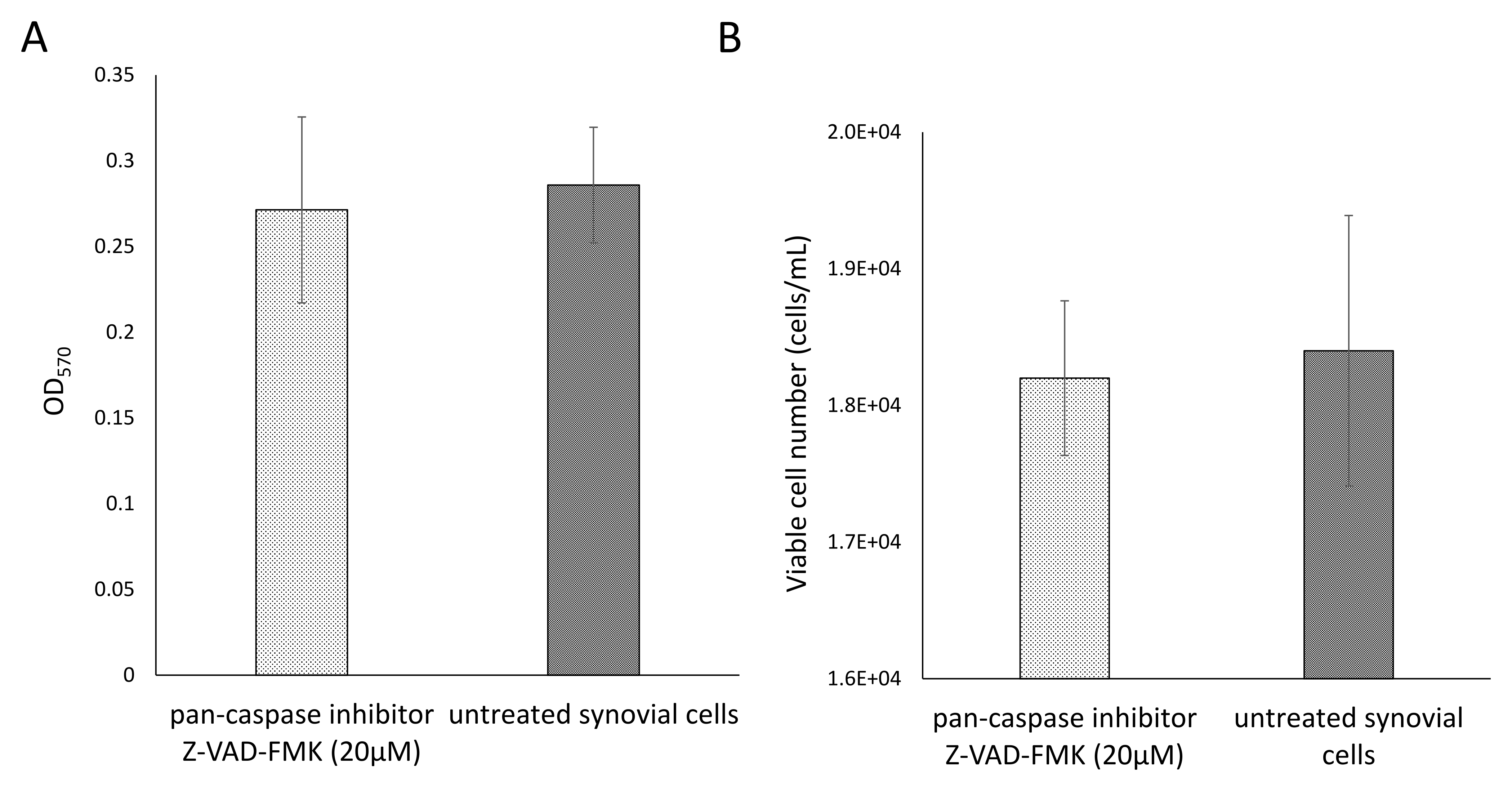

Supplement: Supplementary file 1 [file life-11-01359-s001.zip › Figure S3.tif]
